# Androgen-related sperm storage in oviduct of Chinese Soft-Shelled Turtle *in vivo* during annual cycle

**DOI:** 10.1038/srep20456

**Published:** 2016-02-05

**Authors:** Tengfei Liu, Xiaoya Chu, Yufei Huang, Ping Yang, Quanfu Li, Lisi Hu, Hong Chen, Qiusheng Chen

**Affiliations:** 1 Laboratory of Animal Cell Biology and Embryology, College of Veterinary Medicine, Nanjing Agricultural University, Nanjing, Jiangsu Province, 210095, China

## Abstract

Long-term sperm storage in the female genital tract is essential for the appropriate timing of reproductive events in animals with asynchronous copulation and ovulation. However, the mechanism underlying the prolonged storage of spermatozoa is largely unexplored in turtles. In the present study, the role of androgen in sperm storage was investigated in the oviduct of the Chinese soft-shelled turtle, *Pelodiscus sinensis*. Morphological analysis revealed that spermatozoa were observed in the vagina, uterus and isthmus of the oviduct throughout the hibernation season. The increase of circulating testosterone and dihydrotestosterone levels were consistent with the arrangement of spermatozoa that had their head embedded among the cilia of the oviduct mucosal epithelium. Immunohistochemical analysis revealed that androgen receptor was distributed throughout the cytoplasm of gland cells and among the cilia of ciliated cells. Furthermore, marked variations in protein and mRNA levels of androgen receptor were validated through Western blot and qPCR analyses. The localization and the variation of androgen receptor demonstrated the crucial roles of androgens in sperm storage in the oviduct of *P. sinensis*. These results provide fundamental insights into the interaction of androgen and sperm storage and facilitate the elucidation of the mechanism of sperm storage in turtles.

Sperm storage in the female reproductive tract is defined as the retention of viable spermatozoa for an extended period of time^1^. Long-term sperm storage is used by a variety of animal species, including mammals, insects, fish, birds and reptiles, in which copulation is consistently asynchronous with ovulation^2^,^3^,^4^,^5^,^6^. Moreover, in reptiles, sperm storage has been described in numerous chelonians; the duration of this process is approximately 6 months in the Indian flapshell turtle (*Lissemys punctata punctata*)^7^ and up to 3 years in the painted turtles (*Chrysemys picta*)^8^. Although the prolonged storage of spermatozoa has been demonstrated and the evolutionary significance has also been reported in many turtle species^9^, the mechanisms underlying sperm storage have not been fully elucidated.

Mechanistically, the ejaculated spermatozoa intimately and specifically contact oviduct epithelial cells, a demonstrated characteristic of sperm storage in many reptile species^10^,^11^. Some studies have suggested that oviduct epithelial cells actively provide nutrients for prolonged spermatozoa survival^12^. Furthermore, the interaction of stored spermatozoa with epithelium might protect these sex cells from various degradation factors^13^. Recent studies have suggested that direct contact between spermatozoa and epithelial cells is essential to promote *de novo* gene transcription and synthesis of new proteins in oviduct cells^14^,^15^. Thus, these findings suggest that oviduct epithelial cells provide a suitable milieu to support long-term sperm storage. Understanding the relationship of spermatozoa and epithelial cells is a first critical step to elucidate the mechanism of prolonged sperm storage in turtles.

Previous studies have suggested that the ultimate mechanism underlying sperm storage might involve hormones^16^. An increase in the concentration of circulating androgen has been detected in females of a number of reptile species, including the turtles *Caretta caretta*^17^, *Chrysemys picta*^18^, *Chelonia mydas*^19^ and *Sternotherus odoratus*^20^. Among these species, the androgen concentrations in females were similar to those in males. The levels of circulating androgens may directly determine spermatozoa viability and integrity in many male species^21^,^22^. Moreover, a recent study suggested that androgen creates a unique microenvironment that contributes to prolonged sperm storage in female *Scotophilus heathi* (a tropical vespertilionid bat)^23^. Determining the roles of androgens in female turtles could provide further evidence for dissecting the potential mechanism underlying sperm storage. However, little information is available on the relationship between sperm storage and hormone levels in turtles.

The Chinese soft-shelled turtle (*Pelodiscus sinensis*) is a widely raised ancient reptile species with high edible and pharmaceutical value in China. In *P. sinensis*, spermatogenesis is initiated in early summer (June), and subsequently the mature spermatozoa are released from the epididymis in late autumn (October). Although oogenesis also occurs during the summer, similar to spermatogenesis, ovulation does not occur until the following spring. Thus, for autumn breeding female *P. sinensis*, spermatozoa are stored during the hibernation season (from November to the next April) in the oviduct^24^,^25^,^26^. In previous studies, we clearly demonstrated prolonged sperm storage in the oviduct of female *P. sinensis*^27^,^28^. Furthermore, high levels of circulating testosterone were measured in male *P. sinensis* during the period of active spermatogenesis^29^. Stereological studies also demonstrated that the testosterone concentration was markedly low at the quiescent phase of spermatogenesis^30^. However, the relationship between androgen and sperm storage remains unclear, and there are no reports on seasonal variations in the androgen levels in female *P. sinensis*. The objective of the present study was to investigate the localization and expression of androgen receptor in the oviduct of *P. sinensis* using immunohistochemical (IHC) and Western blot analyses to reveal the roles of androgens in long-term sperm storage. These results will enhance the current understanding of the relationship between androgen and sperm storage and facilitate the elucidation of the mechanism underlying sperm storage in *P. sinensis*.

## Results

### Spermatozoa is stored in the oviduct during the hibernation season

**Figure 1:**
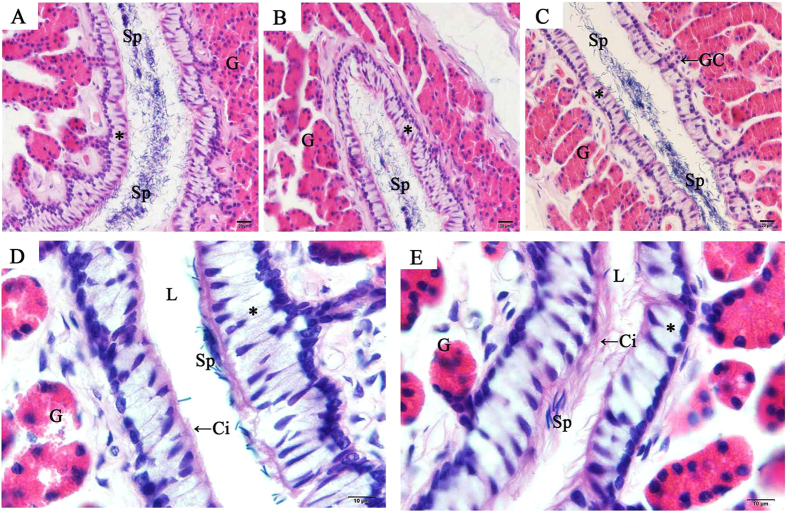
Distribution of spermatozoa in the oviduct of *P. sinensis* during the hibernation season (from November to the next April), H&E staining. (**A**) Vagina in November. (**B**) Uterus in November. (**C**) Isthmus in November. (**D**) In January, spermatozoa were embedded among the cilia in the isthmus. (**E**) In April, spermatozoa were embedded among the cilia in the isthmus. Cilia (Ci), epithelium (*), gland (G), gland conduit (GC), lumen (L), spermatozoa (Sp). Scale bar = 20 μm (**A**–**C**) and 10 μm (**D**,**E**).

**Figure 2:**
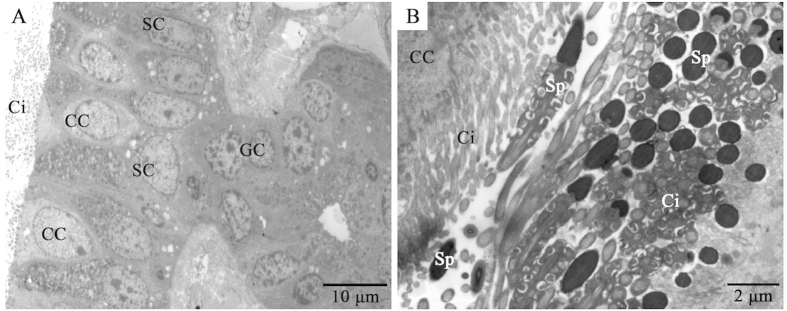
TEM showing the isthmus of the oviduct in *P. sinensis* in January. (**A**) The mucosal epithelium of the oviduct comprises ciliated and secretory cells. (**B**) Many spermatozoa were embedded among the cilia. Secretory cell (SC), ciliated cell (CC), gland cell (GC), cilia (Ci), spermatozoa (Sp). Scale bar = 10 μm (**A**) and 2 μm (**B**).

In female *P. sinensis* during the hibernation season (from November to the next April), spermatozoa were observed in the cavity of the oviduct, including the vagina (Fig. 1A), uterus (Fig. 1B) and isthmus (Fig. 1C). In November, numerous spermatozoa were observed and randomly distributed in the isthmus lumen, while only a few sex cells were in close contact with the epithelial cilia (Fig. 1C). In January, a small amount of spermatozoa were observed in the isthmus lumen (Fig. 1D). These spermatozoa were primarily orientated with the heads towards epithelial cilia in the isthmus (Figs 1D and 2B). These phenomena were also observed in April (Fig. 1E), suggesting that these conditions might consistently persist from early January until the end of hibernation in late April. TEM showed apparent ultrastructural differences between ciliated and secretory cells in the mucosal epithelium (Fig. 2A). The ciliated cells are characterized by abundant cilia on the broad surface. The nuclei of ciliated cells are voluminous and ovoid in shape, and typically medially or basally located. The secretory cells attached to the basal lamina extend to the lumen, and the nuclei are typically oval and basally located.

### Circulating T and DHT concentrations during the hibernation and non-hibernation seasons

**Figure 3:**
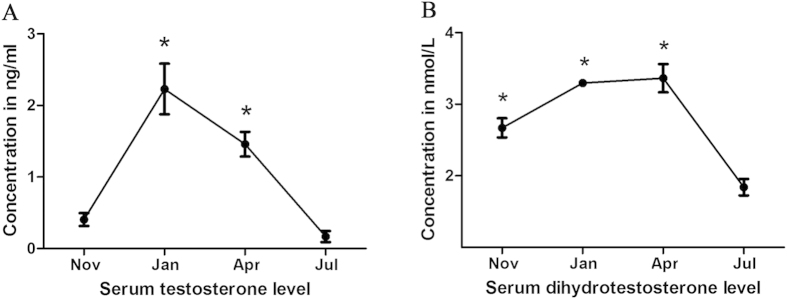
Circulating testosterone (A) and dihydrotestosterone (B) concentrations during the hibernation (from November to the next April) and non-hibernation (July) seasons. For testosterone, the values (*) obtained in January and April were significantly different (*P* < 0.05) from those obtained in July. For dihydrotestosterone, the values (*) obtained in November, January and April were significantly different (*P* < 0.05) from those obtained in July.

In the present study, the concentrations of circulating T (Fig. 3A) and DHT (Fig. 3B) in the serum of female *P. sinensis* were detected at different phases (hibernation and non-hibernation seasons) using ELISA. The results showed that the concentrations of these hormones significantly varied from November to July. In January and April, the circulating T concentration was significantly higher (*P* < 0.05) compared with that in July, and the levels peaked in January. The concentration of circulating DHT was highest in April, followed by January and November, whereas a lower concentration of circulating DHT was observed in July. These observations revealed that the concentrations of circulating T and DHT were relatively higher during the hibernation season (from November to the next April) than during the non-hibernation season (July).

### The dynamic expression of AR mRNA at the sites of sperm storage

**Figure 4:**
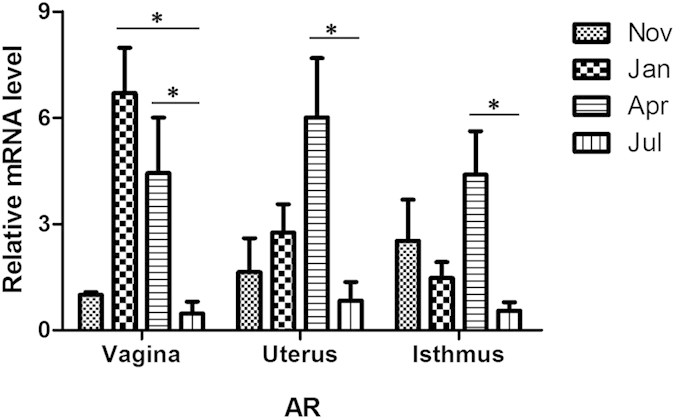
qPCR validation of AR mRNA levels in the isthmus, uterus and vagina of the oviduct in *P. sinensis* during the hibernation (from November to the next April) and non-hibernation (July) seasons. In the vagina, the values (*) obtained in January and April were significantly different (*P* < 0.05) from those obtained in July. In the uterus, the values (*) obtained in April were significantly different (*P* < 0.05) from those obtained in July. In the isthmus, the values (*) obtained in April were significantly different (*P* < 0.05) from those obtained in July.

To validate the roles of AR in sperm storage in female *P. sinensis*, qPCR analysis was used to detect the AR mRNA expression levels in different regions of the oviduct (Fig. 4). The results showed that AR expression varied at different months in certain regions of the oviduct. As expected, the levels of AR mRNA in the vagina were relatively higher in January and April, with lower levels detected in November and July. In uterus of the oviduct, AR expression gradually increased from November to April, peaking in April. In the isthmus, the highest AR expression was observed in April, while the lowest expression was observed in July.

### Localization and expression of AR at sperm storage sites

**Figure 5:**
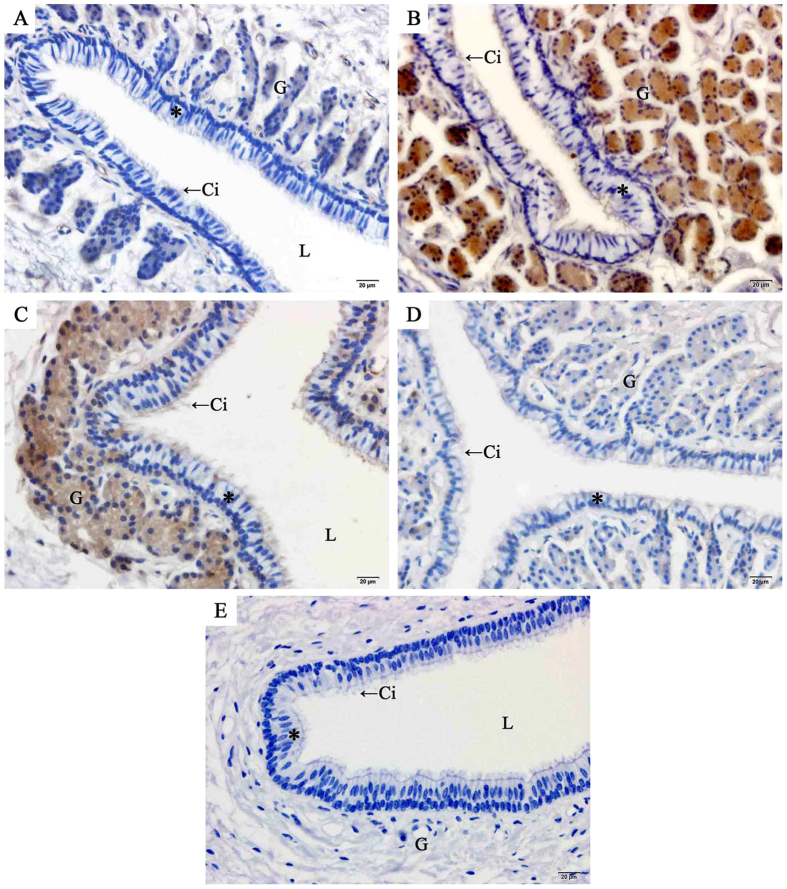
Immunohistochemical localization of AR in the vagina of female *P. Sinensis* during the hibernation (from November to the next April) and non-hibernation (July) seasons. (**A**) November, (**B**) January, (**C**) April, (**D**) July, (**E**) negative control. Cilia (Ci), epithelium (*), gland (G), lumen (L). Scale bar = 20 μm.

**Figure 6:**
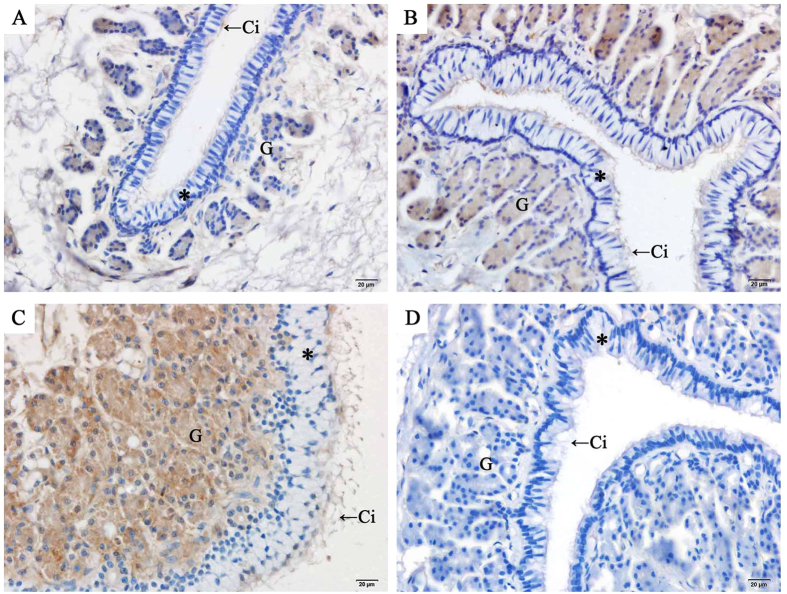
Immunohistochemical localization of AR in the uterus of female *P. Sinensis* during the hibernation (from November to the next April) and non-hibernation (July) seasons. (**A**) November, (**B**) January, (**C**) April, (**D**) July. Cilia (Ci), epithelium (*), gland (G), lumen (L). Scale bar = 20 μm.

**Figure 7:**
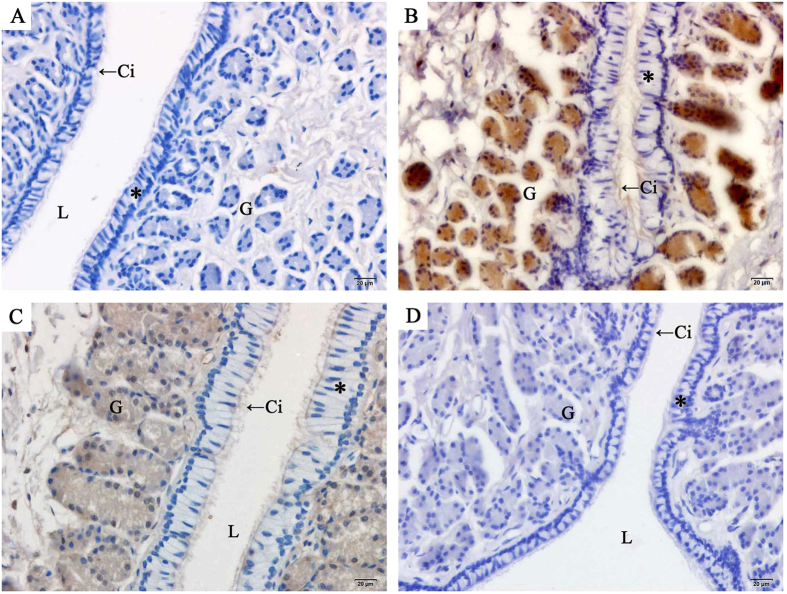
Immunohistochemical localization of AR in the isthmus of female *P. sinensis* during the hibernation (from November to the next April) and non-hibernation (July) seasons. (**A**) November, (**B**) January, (**C**) April, (**D**) July. Cilia (Ci), epithelium (*), gland (G), lumen (L). Scale bar = 20 μm.

The localization of AR in the oviduct of *P. sinensis* was demonstrated through immunohistochemical (IHC) assay (Figs 5, 6, 7). Immunostaining was primarily observed in the cytoplasm of gland cells and the cilia of ciliated cells, whereas no staining was detected in the control sections (Fig. 5E). Moreover, AR expression in different regions of the oviduct revealed similar immunostaining in the vagina (Fig. 5), uterus (Fig. 6) and isthmus (Fig. 7) during the same month. However, variations in immunostaining were observed at different stages (hibernation and non-hibernation seasons), even at different months (November to the next July). For example, in November (early hibernation phase), the gland and ciliated cells in the isthmus showed little immunostaining (Fig. 7A). However, in January (Fig. 7B), gland and ciliated cells in the isthmus showed intense immunostaining. In April (Fig. 7C), the last phase of hibernation, gland cells and ciliated cells also displayed intense AR immunostaining, while during the non-hibernation season (July) (Fig. 7D), markedly little immunostaining was observed in the isthmus.

### Relative expression of AR at different sites of the oviduct

**Figure 8:**
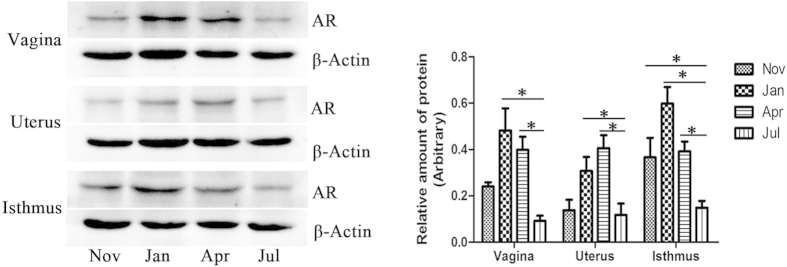
Western blot analysis of AR protein expression in the isthmus, uterus and vagina in the oviduct of female *P. Sinensis* during the hibernation (from November to the next April) and non-hibernation (July) seasons. The histogram represents the densitometric analysis of the immunoblots. In the vagina and uterus, the values (*) obtained in January and April were significantly different (*P* < 0.05) from those obtained in July. In the isthmus, the values (*) obtained in November, January and April were significantly different (*P* < 0.05) from those obtained in July.

Western blotting was performed to validate AR immunoreactivity in the oviduct of *P. sinensis*, with β-actin as an internal control (Fig. 8). The results showed a prominent immunoreactive band at approximately 85 kDa, corresponding to AR protein in three regions of *P. sinensis* oviduct (vagina, uterus and isthmus), and β-actin was observed at approximately 40 kDa. Additionally, the expression analysis revealed that the relative levels of AR protein significantly varied in each region of the oviduct at different months (from November to the next July). In the vagina and isthmus, the amount of AR protein peaked in January and declined from April to July, while the AR protein in the uterus exhibited higher levels in April and January, peaking in April and declining in July. These findings suggested that the levels of AR protein during the hibernation season (November to the next April) were relatively higher compared with those during the non-hibernation season (July), which was consistent with the results of mRNA expression and IHC analyses.

## Discussion

The female reproductive tract plays an important role in the regulation of spermatozoa motility, the storage of spermatozoa prior to fertilization, and the selection of spermatozoa^1^. It has been reported that the sperm storage sites in different reptile species are inconsistent^1^,^31^. In turtles, previous studies have shown that spermatozoa were primarily located in the posterior albumen and the uterine region of the oviduct^32^. However, in the present study, the stored spermatozoa in *P. sinensis* were predominantly observed in the cavity of the oviduct, including the vagina, uterus and isthmus, during the hibernation season (from November to the next April), consistent with previous studies^28^,^33^. It has been reported that intimate contact between spermatozoa and epithelial cell lining is needed for preserving spermatozoa fertility during storage^34^. *In vitro*, binding to the oviduct epithelium prolongs spermatozoa survival and extends the motile life span in some species^35^,^36^. In the present study, the quantity of spermatozoa in the oviduct decreased from the early hibernation (November) to the middle and late stages of hibernation (from January to April), and in January and April, almost all stored spermatozoa were embedded among the cilia of the ciliated cells in the epithelium. The intimate contact between spermatozoa and epithelial cells might play a role in the long-time survival of stored spermatozoa in the oviduct, whereas spermatozoa without intimate contact are eliminated. These findings suggest that the interaction between spermatozoa and oviduct epithelium cells could prolong the life span of stored spermatozoa in *P. sinensis*.

Androgens are essential for sexual development and health throughout the life span of male vertebrates, including the outward development of secondary sex characters and the initiation and maintenance of spermatogenesis^37^. The relationship between androgens and prolonged sperm storage has primarily been studied in males. Jones^22^ reported that the epithelial cells of the cauda epididymis could be stimulated through androgens, thereby ensuring spermatozoa survival for a prolonged period, while the withdrawal of androgens in epithelial cells ultimately resulted in the dissolution and degradation of spermatozoa. In the male lizard (*Sceloporus undulates*), androgens were highly correlated with size and weight of the epididymis, and the stored spermatozoa were lost from regressing epididymis^38^. Furthermore, high circulating levels of androgen were also present in the blood of female reptiles and amphibians during certain periods of their seasonal reproductive cycles^39^,^40^. In the present study, the concentrations of circulating T and DHT were examined in female *P. sinensis* during sperm storage, showing relatively higher levels of these hormones during the hibernation season (from November to the next April) than during the non-hibernation season (July). These observations were consistent with studies in female bats. Indeed, Roy and Krishna^23^ showed that a high concentration of testosterone was necessary to maintain the integrity and viability of stored spermatozoa in the oviduct. These results implied the probable relationship between androgens and prolonged sperm storage in female *P. sinensis*.

Androgens modulate multiple developmental and physiological processes through binding to AR, a member of the nuclear receptor superfamily^41^. The localization of AR in sperm storage would provide evidence for the roles of androgens. In the present study, IHC and Western blot analyses revealed that AR was localized to the vagina, uterus and isthmus of female *P. sinensis*. To the best of our knowledge, this is the first report demonstrating the presence of AR at sites of the prolonged sperm storage in the oviduct of *P. sinensis.* IHC showed that AR was distributed throughout the oviduct epithelium and gland cells. In the oviduct epithelium of *P. sinensis*, there are two primary cell types: ciliated cells and secretory cells^27^. A variety of functions have been attributed to ciliated cells. Some studies have shown that ciliated cells are involved in the movement of mucus and cellular debris in the oviduct^42^. Other studies have suggested that ciliated cells are involved in spermatozoa survival and might be important in spermatozoa transport and ova movement^43^. In the present study, almost all AR immunostaining was observed in the cilia of ciliated cells. Furthermore, H&E staining and TEM analysis demonstrated that spermatozoa were closely attached to epithelial cilia during sperm storage (Figs 1D,E and 2B). These results revealed that androgens might play a role in sperm storage through the cilia. In addition, AR was localized to the cytoplasm in gland cells. Similar cytosolic AR localization has been documented in the oviduct of the turtle *Trachemys scripta*, which also exhibited prolonged sperm storage^44^. Androgens exhibit direct effects mediated through AR to induce receptor dimerization and recruit co-regulators to promote target gene expression^45^,^46^. However, increasing evidence has suggested the physiological effect of non-genomic androgen action^47^,^48^. In many cases, AR is localized outside the nucleus and might trigger biological responses through non-genomic mechanisms. Baron *et al.*^49^ reported that the AR activates the PI3-K/AKT pathway through which androgens could protect epithelial cells against apoptosis. Arvind *et al.*^50^ suggested that treatment with androgens could promote the maturation of oocytes in mice through non-genomic pathways.

Moreover, the results of the present study showed a marked variation in AR protein expression in the vagina, uterus and isthmus of female *P. sinensis* during different stages of sperm storage. IHC and Western blot analyses revealed intense AR expression from January to April during the hibernation season, consistent with the period of peak circulating T and DHT levels. Furthermore, a similar trend of circulating AR mRNA levels was confirmed through qPCR analysis. The presence and variation of AR indicated the vital roles of androgen in sperm storage in the oviduct of *P. sinensis*. It has been suggested that androgens control many signalling pathways mediated through the AR. Previous stereological studies have demonstrated that androgen is responsible for apoptosis in the seminiferous epithelium^30^. A recent study demonstrated that AR protects osteoblasts and osteocytes from apoptosis through the Src/Shc/ERK signalling pathway^51^. Moreover, the involvement of apoptotic pathways in sperm storage has also been reported. Urhausen *et al.*^52^ recently described the expression of apoptosis-related proteins in the dog oviduct, suggesting that the control of apoptosis could be a functional component of sperm storage mechanisms prior to fertilization. Furthermore, a recent study revealed that the oviduct of *P. sinensis* might support sperm storage through anti-apoptosis^33^. Hence, the high AR expression likely indicated that androgens play vital roles in prolonging the spermatozoa storage periods in the oviduct of *P. sinensis*, and androgens are major players in the pathway of sperm storage. More importantly, the mechanism behind sperm storage might be associated with apoptosis, which should be the focus of future studies.

In conclusion, spermatozoa generally congregate in the vagina, uterus and isthmus of female *P. sinensis*. The timing of sperm storage in the oviduct was nearly synchronous with AR expression patterns in glands and cilia. The results of IHC and Western blot analyses strongly suggest an interaction between androgens and sperm storage. Thus, androgens might be crucial players in the regulation of long-term sperm storage, providing a solid foundation for further studies of the mechanism underlying sperm storage in turtles.

## Methods

### Animals

A total of 40 mature female Chinese soft-shelled turtles *P. sinensis*, aged 3–4, were procured from Yangcheng Lake in Suzhou (31°N, 120°E), in the southeast of China. Suzhou has four distinct seasons, in which *P. sinensis* is in the hibernation season from November to the next April and the non-hibernation season from May to October. For each month (November, January, April and July), ten turtles were anaesthetized with an intraperitoneal administration of sodium pentobarbital (20 mg/kg) and were subsequently sacrificed through cervical dislocation. Blood samples were collected, and serums were separated, aliquoted and stored at −70 °C until measurement. One side of the oviduct (vagina, uterus and isthmus) of each turtle was collected and immediately fixed for light and transmission electron microscopy (TEM), respectively. The other side of the oviduct was stored at −70 °C for qPCR and Western blot analyses. The sample procedures were conducted in accordance with the guidelines of the Animal Research Institute Committee of Nanjing Agriculture University. The protocol was approved by the Science and Technology Agency of Jiangsu Province. The approval ID is SYXK (SU) 2010-0005. All efforts were made to minimize animal suffering.

### Haematoxylin-eosin staining

The tissues were fixed in neutral-buffered formalin, embedded in paraffin, and serial sectioned (6 μm). The sections were stained with haematoxylin and counter-stained with eosin (H&E) for observation under a light microscope (Olympus DP73).

### Transmission electron microscopy

The oviduct was removed and was cut into small blocks (1 mm^3^), immersed in a mixture of 2.5% glutaraldehyde fixative in phosphate-buffered saline (PBS) (4 °C, pH 7.4, 0.1 M) for 24 h, followed by fixation in phosphate-buffered 1% osmium tetroxide for 1 h at room temperature. Subsequently, the samples were dehydrated in ascending concentrations of ethyl alcohol, infiltrated with a propylene oxide-Araldite mixture, and embedded in Araldite. Ultra-thin sections (50 nm thickness) were stained with uranyl acetate and lead citrate. The ultrastructure of the oviduct and the spermatozoa were examined and photographed using a JEM-1200EX TEM, and the images were processed using Adobe Photoshop 7.0 software.

### Hormone assay

Serum testosterone (T) and dihydrotestosterone (DHT) were measured through an enzyme-linked immunosorbent assay (ELISA) using a commercially available kit (KA0236, KA1886; Abnova, Walnut, CA, USA). The pooled blood serum of *P. sinensis* were serially diluted and tested against a standard curve. Diluted samples running parallel to the standard curve indicated the validity of this assay in *P. sinensis.* The ELISA was performed according to the manufacturer’s instructions. All samples and standards were measured in duplicate.

### RNA isolation and qPCR analysis

Total RNA was extracted using TRIzol reagent (Invitrogen, Carlsbad, CA, USA) according to the manufacturer’s instructions. The RNA samples were examined using agarose gel electrophoresis stained by GoldView and spectrophotometric analysis. The samples with OD260/280 absorption ratio between 1.8 and 2.0 were used for further analysis. Total RNA samples were reverse-transcribed using SuperScript First-Strand Synthesis System (Invitrogen, Carlsbad, CA, USA). Quantification PCR (qPCR) was performed in a 20-μL volume containing SYBR Green qPCR Supermix (Invitrogen, Carlsbad, CA, USA), 10 mM gene-specific primer and 0.1 mg of cDNA. The reactions were carried out at an initial denaturation step of 95 °C for 10 s, followed by 40 cycles of 95 °C for 5 s, 58 °C for 30 s and 72 °C for 10 s. The melting curve was generated at the end of the PCR run over the range 60–95 °C, increasing the temperature stepwise by 0.5 °C every cycle. Each PCR reaction included a non-template negative control with nuclease-free water instead of cDNA. The relative expression of target genes were normalized to β-actin and analyzed using the delta-delta-CT method. Each experiment was performed in three biological replicates. According to the published gene sequences in NCBI database (AR, XM_006125241.2; β-actin, EU727174.1), specific primers for qPCR were designed using Beacon Designer 7.0 software (Premier Biosoft International, USA). The following primer sequences were used in the present study: AR forward primer (5′-GAAGCCATTGAGCCTATT-3′) and reverse primer (5′-GGAGCAAAGTAAAGCATTC-3′). β-actin forward primer (5′-AGACCCGACAGACTACCTCA-3′) and reverse primer (5′-CACCTGACCATCAGGCAACT-3′).

### Immunohistochemistry

The slides were deparaffinized, hydrated, blocked with 30% H_2_O_2_ in distilled water to eliminate endogenous peroxidase activity, and incubated with boiling buffered citrate. The tissue expression of AR was determined using a primary antibody against AR (ab198394, Abcam Inc., Cambridge, MA, USA) at a 1:250 dilution and the Vectastain Elite ABC Kit (Vector Laboratories, Burlingame, CA) according to the manufacturer’s instructions, followed by visualization using FAST DAB Peroxidase Substrate (Sigma, St Louis, MO, USA). The slides were observed and photographed under a light microscope. The negative controls were obtained after exchanging primary antibodies with PBS.

### Western blot analysis

The samples were homogenized in ice-cold RIPA buffer (25 mM Tris/HCl (pH 7.6), 150 mM NaCl, 1% sodium deoxycholate, 1% Nonidet-P40, 0.1% SDS, and 0.05 mM PMSF). The protein concentration was quantified using a BCA protein assay (Thermo Fisher Scientific, Rockford, USA). Equal amounts of protein (40 μg/lane) were subjected to 8% SDS-PAGE and subsequently transferred to polyvinylidene di-fluoride (PVDF) (Millipore, Bedford, MA) membranes. After blocking in 5% fat-free dry milk, the membranes were incubated overnight at 4 °C with an anti-AR antibody (ab198394, Abcam Inc., Cambridge, MA, USA) diluted 1:1000. After washing, the membrane was incubated with peroxidase-linked goat anti-rabbit IgG (1:5000, BS13278, Bioworld Technology Inc., Louis Park, MN) for 2 h. Bound antibodies were detected using an ECL detection system (Vazyme Biotech, China). The immunoreactive bands were quantified using Quantity One software (Bio-Rad Laboratories). The validity of AR antibody in *P. sinensis* has been detected by negative and positive control analysis.

### Statistical analysis

The data are expressed as the means ± SEM. The data were analysed using SPSS software version 14.0 with one-way ANOVA, followed by Duncan’s test. The differences were considered significant at *P* < 0.05.

## Additional Information

**How to cite this article**: Liu, T. *et al.* Androgen-related sperm storage in oviduct of Chinese Soft-Shelled Turtle *in vivo* during annual cycle. *Sci. Rep.* **6**, 20456; doi: 10.1038/srep20456 (2016).

## References

[b1] Holt W. & Lloyd R. Sperm storage in the vertebrate female reproductive tract: how does it work so well? Theriogenology. 73, 713–722 (2010). 19632711 10.1016/j.theriogenology.2009.07.002

[b2] Pfeiffer B. & Mayer F. Spermatogenesis, sperm storage and reproductive timing in bats. J. Zool. 289, 77–85 (2013).

[b3] Phillips K. P., Jorgensen T. H., Jolliffe K. G. & Richardson D. S. Potential inter-season sperm storage by a female hawksbill turtle. Mar. Turt. Newsl. 140, 13–14 (2014).

[b4] Avila F. W., Mattei A. L. & Wolfner M. F. Sex peptide receptor is required for the release of stored sperm by mated *Drosophila melanogaster* females. J. Insect. Physiol. 76, 1–6 (2015). 25783955 10.1016/j.jinsphys.2015.03.006 PMC4430431

[b5] Bernal M. A. *et al.* Long-erm sperm storage in the brownbanded bamboo shark *Chiloscyllium punctatum*. J. Fish. Biol. 86, 1171–1176 (2015). 25545440 10.1111/jfb.12606

[b6] Hemmings N., Birkhead T., Brillard J., Froment P. & Briere S. Timing associated with oviductal sperm storage and release after artificial insemination in domestic hens. Theriogenology. 83, 1174–1178 (2015). 25638349 10.1016/j.theriogenology.2014.12.022

[b7] Sarkar S., Sarkar N. & Maiti B. Oviductal sperm storage structure and their changes during the seasonal (dissociated) reproductive cycle in the soft-shelled turtle *Lissemys punctata punctata*. J. Exp. Zool. Comp. Exp. Biol. 295, 83–91 (2003). 10.1002/jez.a.10135 12506406

[b8] Pearse D. E., Janzen F. J. & Avise J. C. Genetic markers substantiate long-term storage and utilization of sperm by female painted turtles. Heredity. 86, 378–384 (2001). 11488975 10.1046/j.1365-2540.2001.00841.x

[b9] Pearse D. & Avise J. Turtle mating systems: behavior, sperm storage, and genetic paternity. J. Hered. 92, 206–211 (2001). 11396580 10.1093/jhered/92.2.206

[b10] Sever D. M. & Hamlett W. C. Female sperm storage in reptiles. J. Exp. Zool. 292, 187–199 (2002). 11754034 10.1002/jez.1154

[b11] Siegel D. S. & Sever D. M. Sperm aggregations in female *Agkistrodon piscivorus* (Reptilia: Squamata): a histological and ultrastructural investigation. J. Morphol. 269, 189–206 (2008). 17935196 10.1002/jmor.10588

[b12] Krishna A. The relationship between spermatozoa and epithelium of the female genital tract during sperm storage in the greater yellow bats (*Scotophilus heathi*): the light and electronmicroscopic observations. Proc. Natl. Sci. Counc. Repub. China B. 21, 31–36 (1997). 9208484

[b13] Huang V. W. *et al.* Cell membrane proteins from oviductal epithelial cell line protect human spermatozoa from oxidative damage. Fertil Steril. 99, 1444–1452 (2013). 23312221 10.1016/j.fertnstert.2012.11.056

[b14] Fazeli A., Affara N. A., Hubank M. & Holt W. V. Sperm-induced modification of the oviductal gene expression profile after natural insemination in mice. Biol. Reprod. 71, 60–65 (2004). 14973272 10.1095/biolreprod.103.026815

[b15] Georgiou A. S. *et al.* Modulation of the oviductal environment by gametes. J. Proteome Res. 6, 4656–4666 (2007). 18004800 10.1021/pr070349m

[b16] Crichton E. Reproductive biology of bats. (Academic Press, London, 2000).

[b17] Wibbels T., Owens D. W., Limpus C. J., Reed P. C. & Amoss M. S. Seasonal changes in serum gonadal steroids associated with migration, mating, and nesting in the loggerhead sea turtle (*Caretta caretta*). Gen. Comp. Endocr. 79, 154–164 (1990). 2354777 10.1016/0016-6480(90)90099-8

[b18] Klicka J. & Mahmoud I. The effects of hormones on the reproductive physiology of the painted turtle, Chrysemys picta. Gen. Comp. Endocr. 31, 407–413 (1977). 881114 10.1016/0016-6480(77)90029-6

[b19] Licht P., Wood J., Owens D. W. & Wood F. Serum gonadotropins and steroids associated with breeding activities in the green sea turtle *Chelonia mydas*: I. Captive animals. Gen. Comp. Endocr. 39, 274–289 (1979). 499756 10.1016/0016-6480(79)90122-9

[b20] McPherson R., Boots L., MacGregor R. & Marion K. Plasma steroids associated with seasonal reproductive changes in a multiclutched freshwater turtle, *Sternotherus odoratus*. Gen. Comp. Endocr. 48, 440–451 (1982). 7160611 10.1016/0016-6480(82)90179-4

[b21] Dohle G., Smit M. & Weber R. Androgens and male fertility. World J. Urol. 21, 341–345 (2003). 14566423 10.1007/s00345-003-0365-9

[b22] Jones R. Sperm survival versus degradation in the mammalian epididymis: a hypothesis. Biol. Reprod. 71, 1405–1411 (2004). 15215193 10.1095/biolreprod.104.031252

[b23] Roy V. K. & Krishna A. Evidence of androgen-dependent sperm storage in female reproductive tract of Scotophilus heathi. Gen. Comp. Endocr. 165, 120–126 (2010). 19539620 10.1016/j.ygcen.2009.06.012

[b24] Han X. K., Zhang L., Hei N. N. & Chen Q. S. Sperm storage in male and female soft-shelled turtles, *Trionyx sinensis* in Hibernation. *Journal of Fishery* Sciences of China. 5 (2007).

[b25] Han X. *et al.* Seasonal changes of sperm storage and correlative structures in male and female soft-shelled turtles, Trionyx sinensis. Anim. Reprod. Sci. 108, 435–445 (2008). 17997057 10.1016/j.anireprosci.2007.09.011

[b26] Bian X. *et al.* Ultrastructure of epididymal epithelium and its interaction with the sperm in the soft-shelled turtle *Pelodiscus sinensis*. Micron. 54, 65–74 (2013). 24041582 10.1016/j.micron.2013.08.009

[b27] Han X. *et al.* Ultrastructure of anterior uterus of the oviduct and the stored sperm in female soft-shelled turtle, Trionyx sinensis. Anat. Rec. 291, 335–351 (2008). 10.1002/ar.20649 18231967

[b28] Chen S. *et al.* Sperm storage and spermatozoa interaction with epithelial cells in oviduct of Chinese soft-shelled turtle, *Pelodiscus sinensis*. Ecol. Evol. 5, 3023–3030 (2015). 26357535 10.1002/ece3.1575 PMC4559046

[b29] Mao W. & Wang Z. Seasonal variations of testicular and epididymal structure and plasma levels of testosterone in the soft-shelled turtle. J. Nanjing Normal University (Nat Sci). 2, 53–57 (1997).

[b30] Zhang L., Han X. K., Qi Y. Y., Liu Y. & Chen Q. S. Seasonal effects on apoptosis and proliferation of germ cells in the testes of the Chinese soft-shelled turtle, Pelodiscus sinensis. Theriogenology. 69, 1148–1158 (2008). 18377973 10.1016/j.theriogenology.2008.01.028

[b31] Gist D. & Jones J. Storage of sperm in the reptilian oviduct. Scanning Microsc. 1, 1839–1849 (1987). 3433065

[b32] Gist D. H. & Jones J. M. Sperm storage within the oviduct of turtles. J. Morphol. 199, 379–384 (1989). 10.1002/jmor.1051990311 29865618

[b33] Le Y. *et al.* B-Cell Lymphoma-2 localization in the female reproductive tract of the chinese soft-helled turtle, *Pelodiscus Sinensis* and its relationship with sperm storage. Anat. Rec. (2015). doi: 10.1002/ar.23258. 26285642

[b34] Druart X. Sperm interaction with the female reproductive tract. Reprod. Domest. Anim. 47, 348–352 (2012). 22827391 10.1111/j.1439-0531.2012.02097.x

[b35] Pacey A., Davies N., Warren M., Barratt C. & Cooke L. Hyperactivation may assist human spermatozoa to detach from intimate association with the endosalpinx. Hum. Reprod. 10, 2603–2609 (1995). 8567779 10.1093/oxfordjournals.humrep.a135754

[b36] Apichela S., Jiménez-Díaz M., Roldan-Olarte M., Valz-Gianinet J. & Miceli D. *In vivo* and *in vitro* sperm interaction with oviductal epithelial cells of llama. Reprod. Domest. Anim. 44, 943–951 (2009). 20468081 10.1111/j.1439-0531.2008.01125.x

[b37] McLachlan R. I. *et al.* Hormonal regulation of spermatogenesis in primates and man: insights for development of the male hormonal contraceptive. J. Androl. 23, 149–162 (2002). 11868805

[b38] McKinney R. B. & Marion K. R. Plasma androgens and their association with the reproductive cycle of the male fence lizard, *Sceloporus undulatus*. Comp. Biochem. Phys. A. 82, 515–519 (1985). 10.1016/0300-9629(85)90425-6 2866872

[b39] Moore M. C. & Lindzey J. Biology of the Reptilia. (University of Chicago Press, Chicago, 1992).

[b40] Lind C. M., Husak J. F., Eikenaar C., Moore I. T. & Taylor E. N. The relationship between plasma steroid hormone concentrations and the reproductive cycle in the Northern Pacific Rattlesnake, *Crotalus oreganus*. Gen. Comp. Endocr. 166, 590–599 (2010). 20138180 10.1016/j.ygcen.2010.01.026

[b41] Staub N. L. & Beer D. M. The role of androgens in female vertebrates. Gen. Comp. Endocr. 108, 1–24 (1997). 9378263 10.1006/gcen.1997.6962

[b42] Palmer B. D. & Guillette L. Histology and functional morphology of the female reproductive tract of the tortoise *Gopherus polyphemus*. Am. J. Bot. 183, 200–211 (1988). 10.1002/aja.1001830303 3213826

[b43] Girling J., Cree A. & Guillette L. Oviductal structure in a viviparous New Zealand gecko, Hoplodactylus maculatus. J. Morphol. 234, 51–68 (1997). 10.1002/(SICI)1097-4687(199710)234:1<51::AID-JMOR5>3.0.CO;2-Q 29852640

[b44] Selcer K. W., Smith S., Clemens J. W. & Palmer B. D. Androgen receptor in the oviduct of the turtle, *Trachemys scripta*. Comp. Biochem. Phys. B. 141, 61–70 (2005). 10.1016/j.cbpc.2005.01.013 15820135

[b45] Heinlein C. A. & Chang C. Androgen receptor (AR) coregulators: an overview. Endocr. Rev. 23, 175–200 (2002). 11943742 10.1210/edrv.23.2.0460

[b46] Lee H. J. & Chang C. Recent advances in androgen receptor action. Cell Mol. Life Sci. 60, 1613–1622 (2003). 14504652 10.1007/s00018-003-2309-3 PMC11146054

[b47] Simoncini T. & Genazzani A. R. Non-genomic actions of sex steroid hormones. Eur. J. Endocrinol. 148, 281–292 (2003). 12611608 10.1530/eje.0.1480281

[b48] Foradori C., Weiser M. & Handa R. Non-genomic actions of androgens. Front. Neuroendocrin. 29, 169–181 (2008). 10.1016/j.yfrne.2007.10.005 PMC2386261 18093638

[b49] Silvère B. *et al.* Androgen receptor mediates non-genomic activation of phosphatidylinositol 3-OH kinase in androgen-sensitive epithelial cells. J. Biol. Chem. 279, 14579–14586 (2004). 14668339 10.1074/jbc.M306143200

[b50] Arvind G., Michelle J. & Hammes S. R. Androgens promote maturation and signaling in mouse oocytes independent of transcription: a release of inhibition model for mammalian oocyte meiosis. Mol. Endocrinol. 18, 97–104 (2004). 14576339 10.1210/me.2003-0326

[b51] Kousteni S. *et al.* Nongenotropic, sex-nonspecific signaling through the estrogen or androgen receptors: dissociation from transcriptional activity. Cell. 104, 719–730 (2001). 11257226

[b52] Urhausen C. *et al.* Apoptosis in the uterotubal junction and oviductal isthmus during the estrous cycle of the bitch. Anat. Rec. 294, 342–348 (2011). 10.1002/ar.21300 21235009

